# Emerging strategies to bridge the gap between pharmacogenomic research and its clinical implementation

**DOI:** 10.1038/s41525-020-0119-2

**Published:** 2020-03-05

**Authors:** Volker M. Lauschke, Magnus Ingelman-Sundberg

**Affiliations:** Department of Physiology and Pharmacology, Karolinska Institutet, SE-171 77 Stockholm, Sweden

**Keywords:** Predictive markers, Genetic markers

## Abstract

The genomic inter-individual heterogeneity remains a significant challenge for both clinical decision-making and the design of clinical trials. Although next-generation sequencing (NGS) is increasingly implemented in drug development and clinical trials, translation of the obtained genomic information into actionable clinical advice lags behind. Major reasons are the paucity of sufficiently powered trials that can quantify the added value of pharmacogenetic testing, and the considerable pharmacogenetic complexity with millions of rare variants with unclear functional consequences. The resulting uncertainty is reflected in inconsistencies of pharmacogenomic drug labels in Europe and the United States. In this review, we discuss how the knowledge gap for bridging pharmacogenomics into the clinics can be reduced. First, emerging methods that allow the high-throughput experimental characterization of pharmacogenomic variants combined with novel computational tools hold promise to improve the accuracy of drug response predictions. Second, tapping of large biobanks of therapeutic drug monitoring data allows to conduct high-powered retrospective studies that can validate the clinical importance of genetic variants, which are currently incompletely characterized. Combined, we are confident that these methods will improve the accuracy of drug response predictions and will narrow the gap between variant identification and its utilization for clinical decision-support.

## Introduction

The past several decades of research have seen a dramatic increase in our understanding of how inter-individual genetic variation can impact drug pharmacokinetics, response, and toxicity. However, the clinical application of this knowledge has progressed only slowly. A variety of reasons have been identified to contribute to this latency in clinical implementation.^1–3^ Arguably among the most important factors is the substantial amount of underpowered preliminary studies that report interactions between specific variants and pharmacological phenotypes without replication in independent cohorts or corroborative mechanistic data. Such associations are not useful to guide prescribing and fuel the frequently stated concerns with clinical utility as a key barrier for the clinical use of pharmacogenomics. Partly as a consequence of this vast body of literature, clinicians and service providers feel insecure and insufficiently educated in pharmacogenomics to promote further clinical implementation.

In addition, pharmacogenomic biomarker discovery is complicated by the remarkable pharmacogenetic complexity. Specifically, recent next-generation sequencing (NGS) have identified thousands of novel pharmacogenetic variants that are overlooked when using conventional analysis methods for single nucleotide polymorphism and copy number variation (CNV) profiling.^4,5^ Importantly, many of these rare variants putatively affect the function of the corresponding gene product and are likely to contribute to inter-individual differences in drug disposition and response. Although statistical tests such as burden or variant-component tests provide important frameworks for the analysis of rare variant associations, their suitability for pharmacogenomic applications is limited due to the large number of causal variants whose effects can differ in directionality.^6^ Interpretation of NGS-based data is thus suggested to use a two-pronged approach. Experimental or clinical information is used for variants for which such data are available, whereas the interpretation of uncharacterized variants has to rely on computational predictions. Importantly, it is evident that the success of such a strategy strongly depends on both solid experimental data as well as accurate computational prediction tools. Finally, there is a lack of clear consensus guidelines in product labels and recommendations from different pharmacogenomic expert groups for utilizing genomic data to guide personalized prescribing.

The field of pharmacogenomic biomarker-guided drug therapy has mainly advanced in the field of oncology where drugs are commonly targeted to specific genetic variations in the somatic tumor genome. However, also germline variants can guide the choice and optimal dosing strategy for various chemotherapeutic agents, including fluoropyrimidines or thiopurines. By contrast, the implementation of pharmacogenomics in other therapeutic areas is lagging behind. In order to enhance the reliability and value of predictive pharmacogenomic information in the clinics, there is a need to expand the genetic information retrieved by considering the entire genetic landscape into clinical decision-making and to directly assess the outcome and cost benefit of sequencing-guided therapy. In this matter, retrospective studies based on previous available therapeutic drug monitoring (TDM) data have provided important contributions.

In the following sections, we summarize the state-of-the-art of how to include the entire pharmacogenetic makeup of an individual, including rare variants, into personalized pharmacogenomic advice. Furthermore, we highlight novel strategies and tools to improve the quality of such drug response predictions. Lastly, we provide examples how retrospective analyses of large existing pharmacokinetic TDM data sets can improve the reliability of conclusions regarding the clinical importance of genetic variations.

## How to improve the functional interpretation of rare pharmacogenetic variability

Inter-individual differences in drug response and toxicity are partly due to heritable factors, with common estimates suggesting that the genetic component might explain 20–30% of this variability. Each individual genome contains around 25,000 genetic variations in exons, including 10,000–12,000 missense variants and 100 putative loss-of-function variants.^7,8^ Genes involved in drug absorption, distribution, metabolism, and excretion (ADME) are particularly complex, and analyses of whole exome and whole genome sequencing data from more than 100,000 individuals indicated that *CYPs*^9,10^ and drug transporters of the *SLC*^11^ and *SLCO*^12^ gene families harbor tens of thousands of different single nucleotide variants (SNVs) and indels. Importantly, the overwhelming majority of these variants (>98%) are rare with minor allele frequencies in the general population below 1%.^13–16^ Moreover, analyses of CNVs in ADME genes revealed that of the 208 genes analyzed, 201 (97%) carried deletions and duplications, most of which were rare, spanning one to multiple exons.^17^ Similarly, rare SNVs and CNVs were highly prevalent in genes encoding G-protein-coupled receptors, one of the most important families of drug targets.^18^

The identification of rare variants requires the use of methods that can comprehensively profile genes of importance for drug pharmacokinetics and response. Genetically mediated modulation of pharmacokinetic profiles can furthermore translate into altered risks for adverse drug reactions, particularly for chemotherapeutic drugs. Specific examples include variants in *DPYD* that increase fluorouracil toxicity and variations in *TPMT* and *UGT1A1*, which predispose to thiopurine- and irinotecan-induced myelosuppression, respectively. The increase in speed and accuracy of NGS, combined with decreasing sequencing costs, facilitates the use of sequencing for routine applications. Importantly, however, a considerable number of genes with importance for drug response and toxicity (up to 50, including *CYP2D6*, *CYP2A6*, and *HLA* genes) are located in complex loci that feature low complexity regions, segmental duplications, and variable numbers of tandem repeats. Analysis of the genetic variability within such genes requires the use of specialized sequencing technologies and variant calling pipelines. Promising emerging methods in this space include single-molecule real-time sequencing and Nanopore sequencing, which have both been successfully used for whole genome sequencing applications,^19,20^ as well as for targeted haplotyping and CNV profiling of the complex *CYP2D6*^21–24^ and *HLA* loci.^21,25^

To characterize the functional effects of common genetic variants, a variety of methods and tools are available, including genetic association studies, in vitro characterizations, and computational predictions. However, for multiple reasons this toolbox is not directly applicable for rare variant assessments:

i. Genetic association studies require a substantial number of variant carriers to be sufficiently powered to identify effects of a variant of interest on a clinical phenotype, which, depending on the experimental design and phenotypic heterogeneity, can require tens to hundreds or thousands of individuals. However, recruitment of these numbers of carriers for rare alleles is most often unfeasible or impractical.
ii. Functional characterization of tens of thousands of variants using heterologous expression systems is not feasible using conventional protocols.
iii. Most computational algorithms to predict the functional consequences of genetic variants have been trained on pathogenic data sets and use, at least in part, evolutionary conservation as the basis for decision-making. However, for genes involved in drug disposition, evolutionary constraint is generally low and thus conservation at the nucleotide level constitutes only a poor proxy for functional consequences of the encoded gene product.

Importantly, in the last few years a variety of methodological and technological advances have opened exciting avenues to overcome these challenges. In the following two sections, we will provide an overview of computational (“Computational analysis of pharmacogenomic variation”) and experimental (“Experimental high-throughput characterization of pharmacogenomic variants”) strategies to improve the functional interpretation of rare pharmacogenetic variants.

### Computational analysis of pharmacogenomic variation

The translation of personal genomic information into actionable advice requires predictions about the functional consequences of the identified genetic variability for which no experimental or epidemiological characterization data are available. In the past decades, more than 60 different prediction tools have been developed.^26^ SIFT,^27^ PolyPhen2,^28^ PROVEAN,^29^ and MutationTaster2^30^ constitute the most widely used models. However, they are restricted to the interpretation of variants that cause amino acid exchanges. Prediction tools that are suitable for the interpretation beyond missense variants include FATHMM,^31,32^ CADD,^33^ and Eigen.^34^

The vast majority of tools are based on machine learning, often used synonymously with artificial intelligence, to optimize predictions. Training of such models relies on data sets of deleterious and neutral variants in which variations are annotated regarding their functional consequence. As deleterious data sets, most studies use variants implicated in Mendelian disorders, mined from publically available collations such as OMIM, HGMD, or UniProtKB. For neutral variants, the most common approach is to consider all variants as neutral whose frequency is above a certain threshold (e.g., 1% or 5%) in the general population, as revealed by large-scale sequencing projects, such as the 1000 Genomes Project. This selection is based on the rationale that common variants that are present in a considerable fraction of the population are apparently not sufficient to cause clinically manifest heritable disease. From these training sets, the algorithm aims to identify patterns of statistical associations among features, including information about the sequence context of the affected nucleotide, evolutionary conservation, or physicochemical consequences of the amino acid exchange on protein level, and, based on these rules, predict the functional consequence of unannotated variants.

Importantly, although this approach is promising for the prediction of a variant’s pathogenicity, this approach is linked to multiple problems when the functionality of pharmacogenomic variants should be analyzed. First, pharmacogenes harbor a multitude of variants that alter function without being pathogenic. Common examples of variations with minor allele frequencies > 10% that reduce gene function and have established effects on drug pharmacokinetics or toxicity include CYP2C9*2 (rs1799853), CYP2C19*2 (rs4244285), CYP2D6*4 (rs3892097), CYP3A5*3 (rs776746), UGT1A1*28 (rs34983651), and SLCO1B1*5 (rs4149056). Using the approach illustrated above, all these variants are included in the neutral training set. Second, the statistical associations between features are different between pathogenic and functional but non-pathogenic variants. In particular, evolutionary conservation is different between variants within genes associated with congenital diseases that are under purifying selection and those which are not disease associated, such as most pharmacogenes.^35^ As a consequence, application of the relationships between conservation scores and functional impacts that were established on pathogenic vs. non-pathogenic training sets is not suitable to provide reliable predictions in the context of pharmacogenes.

To overcome these limitations, we recently developed a computational tool specifically tailored towards the functional interpretation of pharmacogenetic variations. To this end, we leveraged quantitative, experimentally determined high-quality activity data from 337 variants distributed across 43 genes involved in drug metabolism and transport as training data.^36^ In a first step, we optimized the parameterization of 18 current prediction methods and then integrated the best performing methods into one ensemble score. Using cross-validations, the resulting score achieved sensitivity and specificity of 93% for loss-of-function and functionally neutral variants, respectively, and, importantly, can provide quantitative estimates of the functional consequences of a pharmacogenetic variant. Notably, this predictive power was achieved despite the low number of pharmacogenomic variants available for model training. We expect that model accuracy will further improve as the number of functionally characterized variants increases and in the following section we highlight methods that could be instrumental in this process.

### Experimental high-throughput characterization of pharmacogenomic variants

Due to the sheer number of variants, experimental characterization, the gold standard for functionality assessment, of the entire portfolio of rare pharmacogenetic variants has not been feasible. As a result, the impact of rare variants encountered in the genome of a given patient can currently only be estimated using computational tools, which impairs personalized medicine. Deep mutational scanning constitutes a state-of-the-art technology that combines large-scale parallel mutagenesis with phenotypic selection and deep sequencing to assess the activities of more than 100,000 mutant versions of a protein in a single experiment.^37^

**Fig. 1:**
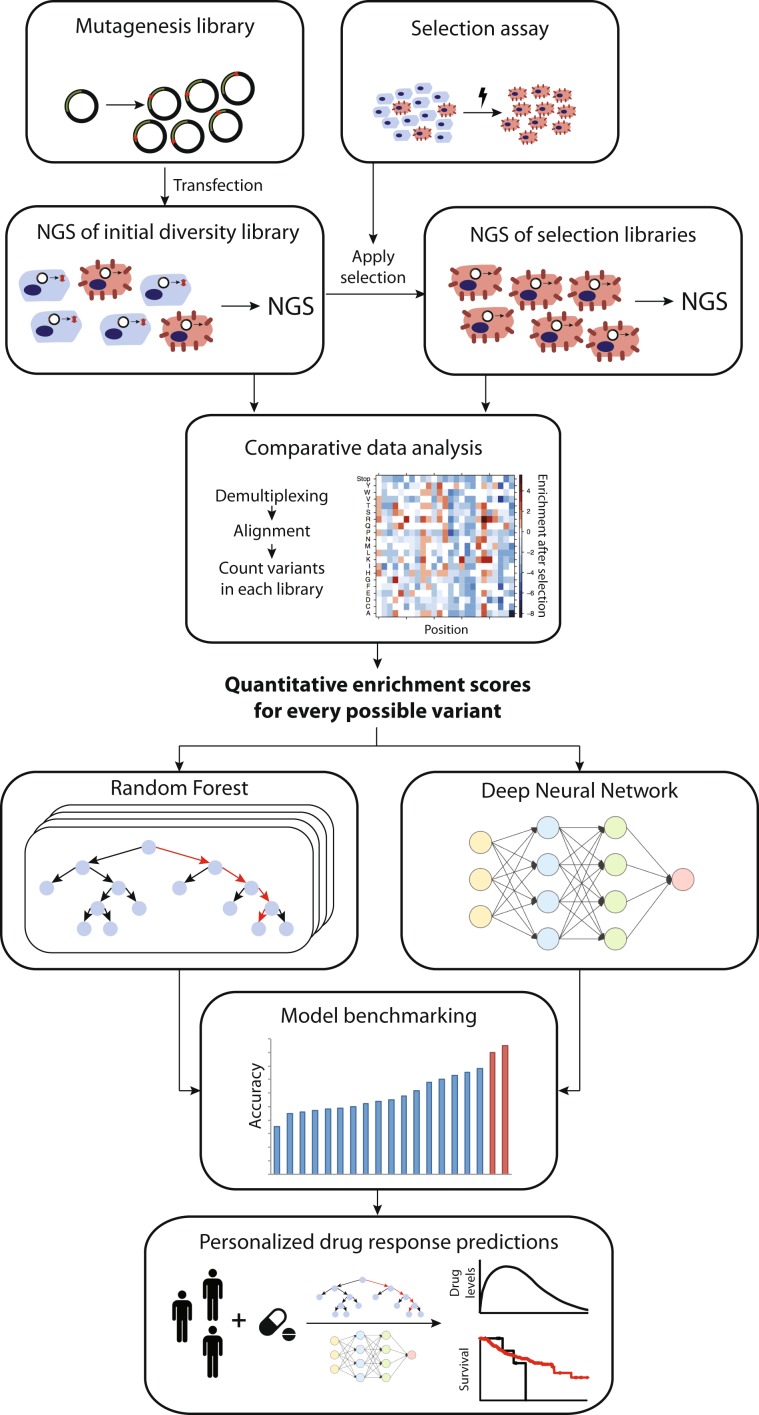
Schematic depiction of how deep mutational scanning aspires to improve the translation of pharmacogenomic information into actionable advice. Cells are transfected with a diversity library of expression plasmids covering many (ideally all possible) amino acid substitutions for a given pharmacogene of interest and a protein-specific selection assay is applied. Deep sequencing of the transfected cells before and after selection, and comparative analysis of the obtained data can provide enrichment scores for each variant, thus enabling the massively parallelized experimental characterization of thousands of variations. Importantly, this data provides a powerful resource for the training and optimization of computational algorithms, including but not restricted to random forests and deep neural networks, which in turn can be applied to the integrated analysis of a patient’s entire pharmacogenome.

Principally, deep mutational scanning requires a cell-based selection assay as well a diversity library of plasmids in which every amino acid is substituted individually to every other amino acid (Fig. 1). Although the selection assay is highly protein specific, metabolic activation of a substrate into a toxic metabolite or transport of toxins into or out of cells is likely applicable in the context of pharmacogenes. Diversity libraries can be constructed using parallelized oligonucleotide-directed mutagenesis, which avoids the bias in the introduction of mutations that occur when using alternative mutagenesis methods, such as error-prone PCR.^38^ One-pot saturation mutagenesis offers an elegant and efficient method for the construction of comprehensive mutagenesis libraries within few days with minimal hands-on time.^39^ The assembled library is then transfected into cells in amounts that result in transformants only harboring a single variant. Subsequently, the selection assay is applied, resulting in the specific selection of transfectants with neutral or deleterious variant, depending on the selection assay configuration. For instance, if deep mutational scanning is used to characterize a drug transporter and the selection assay comprises the export of cytotoxic compounds, variants that reduce the transport activity will be depleted after selection, whereas the frequency of neutral variants will increase accordingly. Variants detected by NGS in the initial diversity library that are over- or underrepresented following selection indicate increased and reduced function variants, respectively. Moreover, the extent of variant representation in the pool directly correlates with resistance to the applied agent, thus providing a quantitative readout of variant function.

This method has been successfully used for the comprehensive evaluation of variant effects on organismal fitness as well as for domain characterizations and the mapping of protein interaction sites, primarily in bacteria, yeast, and rodent.^40–43^ Recently, these analyses were also extended to disease-associated human proteins, including BRCA,^44^ YAP,^45^ and PPARG,^46^ thus providing an appealing methodology to experimentally characterize the entire catalog of possible genetic variability affecting a protein’s amino acid sequence for a given human gene. Notably, however, the workflow has to be tailored to each gene individually, resulting in the protocol to be work- and cost-intensive.

To overcome this difficulty, Matreyek et al.^47^ developed VAMP-Seq, which systematically probes the effects of all variants on protein abundance rather than function per se. Using the clinically actionable genes *PTEN* and *TPMT* as targets, the authors identified 1138 and 777 variants that alter protein levels, respectively. For those PTEN variants for which abundance scores could be obtained, VAMP-Seq correctly flagged 64% of pathogenic variants as low abundance and 12% as possibly low abundance. However, the specificity of VAMP-Seq could not be accurately quantified due to a lack of *PTEN* variants with confirmed functional neutrality. For TPMT, the well-characterized alleles TPMT*2 and TPMT*3 account for 95% of *TPMT* loss-of-function variants and, thus, the immediate clinical impact of the additional variants affecting TPMT abundance is likely limited.

Importantly, such comprehensive functionally annotated data sets provide powerful tools that can be leveraged to improve computational predictions of genes not yet covered by deep mutational scanning. An interesting recent seminal study provided proof-of-concept that training of a decision tree-based model using available large-scale mutagenesis data resulted in superior performance compared with pathogenicity-trained variant predictors.^48^ Specifically, the authors used a total of 21,026 experimentally determined variant effect scores from eight proteins to train a supervised, stochastic gradient boosting learning algorithm. The resulting tool, termed Envision, could predict quantitative variant effects and outperformed the previous predictors SIFT, PolyPhen2, SNAP2, and EVmutation also on protein data that were not used for model fitting. Interestingly, structural features were found to contribute more to model performance compared with evolutionary features, indicating the importance of feature completeness. As expected, inclusion of data from more proteins increased Envision’s predictive performance. However, the tool has not yet been used for systematic pharmacogenetic predictions.

These developments are paralleled by recent advances in the variant effect prediction using deep learning. By leveraging variation data from non-human primate genomes for model training, deep neural networks have been successfully used to predict pathogenic variation in rare disease patients.^49^ Furthermore, training of deep neural networks using available genome-wide association study data or phenotype similarity metrics can facilitate the identification of causative pathogenic variation within and outside of the coding regions.^50,51^ Although the abovementioned tools focus on the identification and interpretation of disease-associated variation, the application of deep learning for pharmacogenomic variant effect prediction is currently limited to specific applications, such as the prediction of metabolic activity of *CYP2D6* genotypes and haplotypes, primarily due to the scarcity of available training data.^52^

For the near future, we foresee that the integration of pharmacogene-specific experimental characterizations with model training based on the resulting quantitative data provides a promising strategy to drastically improve the quality of functional predictions, which constitutes one of the key challenges of personalized pharmacogenomics (Fig. 1).

## How to increase the reliability of pharmacogenomic studies

The low power and reliability of many studies in the published pharmacogenomic literature constitutes a key bottleneck for the translation of pharmacogenomic markers into clinical practice. The most important shortcomings are the limited power, often caused by a low number of patients in relation to the frequencies of the genetic variants studied, unbalanced inclusion and erroneous definition of the patients cohorts studied, analytical bias due to a nonrandomized selection of the patient groups, and lack of result validation in independent cohorts. The resulting uncertainty is reflected in the limited consensus between pharmacogenomic labels as considered by different regulatory agencies.^53,54^

Large prospective randomized clinical trials would be most informative to cope with these problems. However, there is very limited public funding for such pharmacogenomic trials and only few examples without corporate sponsorship have been reported in the literature, such as the EU-PACT study for warfarin gene–dose relationships.^55^ Thus, the field has tapped into the vast resource of available TDM data to analyze the impact of pharmacogenomic variation. Specifically, patients who were treated in the absence of genotypic information and whose drug or metabolite levels were monitored can be retrospectively genotyped, given that appropriate ethical approval is available or can be obtained.

**Fig. 2:**
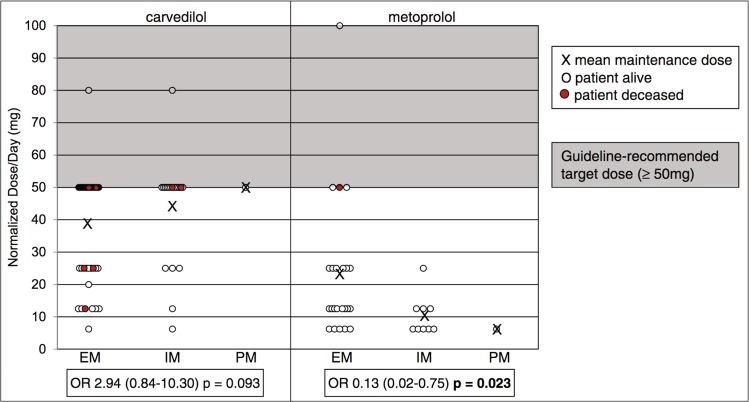
Associations of *CYP2D6* genotype with the achievement of guideline-recommended target doses for carvedilol and metoprolol. Analysis comprises 98 systolic heart failure patients. Only CYP2D6*4 was considered for the definition of CYP2D6 metabolizer status. Patients were 7.7 times more likely to be treated with lower maintenance doses of metoprolol for each CYP2D6*4 allele. For carvedilol, a trend was observed between CYP2D6*4 and higher maintenance dose. Gray shaded box indicates the range of guideline-recommended target doses (≥50 mg). EM extensive metabolizer (no *4 alleles), IM intermediate metabolizer (one *4 allele), OR odds ratio, PM poor metabolizer (two *4 alleles). Figure modified with permission from ref. ^56^.

Interesting examples that demonstrate the power of this design include the relationship of metoprolol maintenance dose in heart failure patients with *CYP2D6* genotype^56,57^ (Fig. 2). Similarly, in a study of 993 patients it was concluded that the dose-adjusted phenytoin blood concentrations were higher and the risk for neurologic side effects was elevated by 2.4-fold among carriers of reduced function *CYP2C9* alleles.^58^ A similar retrospective approach has been applied in many studies from different countries regarding the relative importance of *VKORC1* and *CYP2C9* genotypes for warfarin maintenance doses.^59^ Indeed, a multitude of dosing algorithms have been developed in different countries for such predictions.

Large retrospective studies have been furthermore useful for evaluating gene–dose effects of antidepressant and antipsychotic treatment. In a study encompassing 2087 patients, a correlation between the *CYP2C19* genotype and TDM-based concentrations of escitalopram and metabolites were found, showing that poor and ultrarapid CYP2C19 metabolizers received escitalopram doses above or below the therapeutic interval, respectively.^60^ As a consequence, the rate of drug switching, as a proxy for lack of efficacy or adverse reactions, among these phenotype groups, which affected a total of 10% of patients, was 2.3- to 3-fold higher than in extensive metabolizers.

**Fig. 3:**
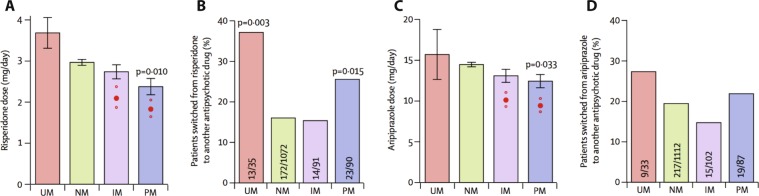
Effect of *CYP2D6* genotype status on risperidone and aripiprazole dose and treatment failure rates. Plots show the associations between CYP2D6 genotype and daily dose and switching frequency for 725 patients treated with risperidone (**a**, **b**) and 890 patients treated with aripiprazole (**c**, **d**). Considered genotypes include CYP2D6*3, *4, *5, *6, *9, *10, *41, and functional duplicated alleles *1xN or *2xN. In **a** and **c**, the dose reductions needed to compensate for the increase of risperidone exposure in patients who were intermediate metabolizers (IM) and poor metabolizers (PM; 95% CI) are indicated by red dots. Error bars indicate SEM. Statistical tests of subgroups are performed using the normal metabolizers (NM) as reference. IM intermediate metabolizers, PM poor metabolizers, UM ultrarapid metabolizers. Figure modified with permission from ref. ^61^.

Similar associations were observed among patients receiving the antipsychotics risperidone or aripiprazole with both poor and ultrarapid CYP2D6 metabolizers being more prone to drug switching than extensive metabolizers.^61^ Interestingly, it was observed that patients carrying reduced activity variants in *CYP2D6* were indeed prescribed lower doses than those with extensive metabolism, despite the fact that the physicians were not aware of the genotype of the patients, indicating that phenotypic peculiarities linked to the drug concentration were noticed by the physician and doses were adjusted accordingly. Interestingly, however, for risperidone these dose adjustments only resulted in 50% of the optimal dose reduction predicted by the *CYP2D6* genotype (Fig. 3).

Overall, these results indicate that the retrospective genotyping of large TDM cohorts can indeed provide with clinically useful information regarding gene drug interactions, which in turn can inform genotype-guided dosing in clinical routine.

## Conclusions

The limited number of sufficiently powered and controlled pharmacogenomic association studies in combination with a lack of functional characterization data for many thousands of genetic variations impair our ability to fully harness a patient’s pharmacogenomic information for genotype-guided prescribing. Thus, although progress in NGS technologies has unlocked exciting possibilities to comprehensively profile pharmacogenomic variability, the resulting additional information is currently not clinically actionable. Novel experimental and computational methods hold promise to improve the functional interpretation of pharmacogenomic complexity. In addition, various companies develop high-throughput expression methods for functional characterizations of missense mutations.

Due to impracticalities involved in recruitment of a sufficient number of rare variant carriers and the associated high cost, we do not envision that assessments of the functional importance of rare genetic variants can be conducted using conventional clinical trial designs. Rather, we envision that retrospective analysis of the numerous large TDM-based data sets with thousands of individuals can increase the possibility to validate potential pharmacokinetic phenotype–genotype relationships and to discover novel associations.

Importantly, pharmacogenomic variability differs drastically across ethnicities. Clinically relevant examples include common reduced function variants in *CYP2B6*, *CYP2C8*, and *CYP2C9*, which are up to 20-fold less prevalent in East Asian populations compared with Africans, whereas East Asians carry the highest frequency of decreased function variants in *CYP2D6* and *CYP1A2*.^62^ Besides these common well-characterized alleles, rare pharmacogenomic variants show similar patterns of inter-ethnic variability. By analyzing exome data from 6503 individuals with European or African ancestry, we previously found that <9% of all rare variants were found in both populations.^13^ Similarly, in an analysis of 141,456 individuals from 7 major human populations, up to 83% of variants in *SLC* and *SLCO* genes were restricted to a single population.^11,12^ These inter-ethnic differences have important clinical implications, as exemplified by the inter-ethnic differences in anticoagulant and antihypertensive dose requirements,^63^ improved gefitinib response rates in East Asians,^64^ increased rates of cutaneous ADRs upon carbamazepine in South and East Asians,^53^ as well as efavirenz-related ADRs in Zimbabwe.^65^ Furthermore, 26 drugs approved by the Food and Drug Administration from 2008 to 2013 carry labels for racial or ethnic differences.^66^ These examples demonstrate that consideration of the specific genetic makeup of the population in question in the framework of a precision public health strategy provides appealing opportunities to improve clinical outcomes. African and admixed Latin American populations are especially heterogeneous and are genetically variable, and, as a result, members of these racial and ethnic groups might benefit most from high-resolution pharmacogenomic characterizations and genetically informed prescribing, particularly in those regions of the world where access to individual genomic profiling is limited.

In conclusion, we think that the gap between identification and utilization of genetic variants for truly individualized treatment recommendations will diminish in the future. This is due to more frequent use of large patient cohorts, which allow firm conclusions regarding the clinical importance of genetic variations, in parallel to the impressive development in analytical techniques and computational functionality predictions of novel variations.
